# Interactive effects between *CDHR3* genotype and rhinovirus species for diagnosis and severity of respiratory tract infections in hospitalized children

**DOI:** 10.1128/spectrum.01181-23

**Published:** 2023-09-26

**Authors:** Yu P. Song, Man F. Tang, Agnes S. Y. Leung, Kin P. Tao, Oi M. Chan, Gary W. K. Wong, Paul K. S. Chan, Renee W. Y. Chan, Ting F. Leung

**Affiliations:** 1 Department of Paediatrics, The Chinese University of Hong Kong, Prince of Wales Hospital, Hong Kong, China; 2 Hong Kong Hub of Paediatric Excellence, The Chinese University of Hong Kong, Hong Kong, China; 3 The Chinese University of Hong Kong-University Medical Center Utrecht Joint Research Laboratory of Respiratory Virus and Immunobiology, The Chinese University of Hong Kong, Hong Kong, China; 4 Department of Microbiology, The Chinese University of Hong Kong, Prince of Wales Hospital, Hong Kong, China; Chinese Academy of Sciences Wuhan Institute of Virology, Wuhan, China

**Keywords:** CDHR3, children, gene-environment interaction, respiratory tract infection, rhinovirus, wheezing

## Abstract

Rhinovirus (RV) is the leading pathogen causing childhood wheezing, with rhinovirus C (RV-C) species reported to cause asthma exacerbation. Allele A of single-nucleotide polymorphism (SNP) *CDHR3*_rs6967330 upregulates epithelial expression of RV-C receptors which results in more severe asthma exacerbations in children. Nevertheless, there are limited data on interactions between *CDHR3* variants and their impact on severity of RV-related pediatric respiratory tract infections (RTIs). Medical records of RV-related RTIs in children aged below 18 years who were hospitalized in two public hospitals in 2015–2016 were independently reviewed by two paediatricians. Archived nasopharyngeal aspirates were retrieved for RV detection and sequencing as well as *CDHR3* genotyping. HaploView v.5.0 and generalized multifactor dimensionality reduction (GMDR) analysis were employed for haplotypic assignment and gene-environment interaction analyses. Among 1019 studied cases, our results confirmed the relationship between RV-C species and more severe RTIs. Besides the top risk variant rs6967330-A, we identified rs140154310-T to be associated with RV-C susceptibility under the additive model [odds ratio (OR) 2.53, 95% CI 1.15–5.56; *P* = 0.021]. Rs140154310 was associated with wheezing illness (OR 2.38, 95% CI 1.12–5.04; *P* = 0.024), with such association being stronger in subjects who wheezed due to RV-C infections (OR 2.71, 95% CI 1.32–5.58; *P* = 0.007). Haplotype GAG constructed from rs4730125, rs6967330, and rs73195665 was associated with increased risk of RV-C infection (OR 1.71, 95% CI 1.11–2.65; *P* = 0.016) and oxygen supplementation (OR 1.93, 95% CI 1.13–3.30; *P* = 0.016). GMDR analyses revealed epistatic interaction between rs140154310 and rs6967330 of *CDHR3* for RV-C infection (*P* = 0.001), RV-C-associated lower RTI (*P* = 0.004), and RV-C-associated wheeze (*P* = 0.007). There was synergistic gene-environmental interaction between rs3887998 and RV-C for more severe clinical outcomes (*P* < 0.001). To conclude, rs140154310-T is another risk variant for RV-C susceptibility and more severe RTIs. Synergistic epistatic interaction is found between *CDHR3* SNPs and RV-C for RTI severity, which is likely mediated by susceptibility to RV-C. Haplotypic analysis and GMDR should be included in identifying prediction models of *CDHR3* for childhood asthma and RTIs.

**IMPORTANCE:**

This case-control study investigated the interaction between CDHR3 genotypes and rhinovirus (RV) species on disease severity in Hong Kong children hospitalized for respiratory tract infection (RTI). There were synergistic effects between RV-C and CDHR3 SNPs for RTI severity, which was mainly driven by RV-C. Specifically, rs6967330 and rs140154310 alone and their epistatic interaction were associated with RV-C-related and severe RTIs in our subjects. Therefore, genotyping of CDHR3 SNPs may help physicians formulate prediction models for severity of RV-associated RTIs.

## INTRODUCTION

Wheezing is a major cause for pediatric hospitalization, and early-life wheezing is commonly the first presentation of childhood asthma. Viral infection is the leading cause of acute wheeze, whereas human rhinovirus (RV) is one of the most commonly detected pathogens in children hospitalized for wheezing illness as well as the main trigger for asthma exacerbation (AE) (1, 2). Infants suffering from RV-induced wheeze are at risk of recurrent wheezing and later asthma development (3). Among RVs, the rhinovirus C (RV-C) species is associated with more severe wheezing phenotypes compared to the A and B species (4, 5). The asthma risk allele rs6967330-A on *CDHR3* was related to the susceptibility to RV-C infection as the encoded variant Tyr529 was associated with higher surface expression of RV-C-specific receptor (6 – 8). The COPSAC2010 and COAST birth cohorts also supported this risk allele to be associated with more frequent respiratory tract infections (RTIs) within the first three years of life and particularly due to RV-C (9). Comparatively, there were limited data on other single-nucleotide polymorphisms (SNPs) of *CDHR3* and their roles in RV-C-associated RTIs. We hypothesized that other SNPs of *CDHR3* also played significant roles in determining susceptibility and severity of RV-C infection. This cross-sectional study investigated the association between *CDHR3* SNPs and severity of RV-C infection among Hong Kong children hospitalized for RTIs.

## RESULTS

### Demographics, RV infections, and clinical outcomes

Overall, 1,564 archived NPAs were retrieved and 1,523 of them had successful genotyping for both host *CDHR3* SNPs and RV species. RV sequencing yielded 48.6% RV-C, 45.7% RV-A. and 5.7% RV-B. The minor allele frequency (MAF) for *CDHR3_*rs6967330 was 9.7% (GG 81.9%, AG 16.9%, and AA 1.2%).

To minimize biased assessment of RTI severity, subjects with clinically significant co-morbidities or respiratory co-infections were excluded (Fig. S1). Table S1 describes the demographics and *CDHR3* genotype distribution in analyzed and excluded subjects. Interestingly, RV-B cases had higher rate of respiratory co-infections than RV-A and RV-C (44% vs 21.5% vs 17.3%; Table S2).

Among 1019 patients involved in genetic analyses, 59.2% were infected with RV-C and 47.6% of cases had lower RTIs. Compared to the other two species, patients infected with RV-C more likely suffered from wheezing illness (odds ratio [OR] 3.49, 95% CI [CI] 2.68–4.54; *P* < 0.001; Table 1). RV-C cases also more likely required oxygen supplement (OR 4.04, 95% CI 2.59–6.30; *P* < 0.001) and systemic corticosteroid treatment (OR 4.23, 95% CI 2.88–6.20; *P* < 0.001).

**TABLE 1:** Relationship between RV-C infection and risks for wheezing illness and RTI severity^*c*^

	RV-A or B (*N* = 518) *n* (%)	RV-C (*N* = 501) *n* (%)	OR/B^*a*^ (95% CI)	*P* value
Diagnosis				
Upper RTI	341 (65.8)	193 (38.5)	0.32 (0.24–0.41)	**<0.001^*d*^**
Lower RTI	177 (34.2)	308 (61.5)	3.16 (2.44–4.09)	**<0.001**
Wheezing illness^*b*^	153 (29.5)	295 (58.9)	3.49 (2.68–4.54)	**<0.001**
Asthma exacerbation	74 (14.3)	182 (36.3)	3.96 (2.86–5.49)	**<0.001**
Pneumonia	24 (4.6)	13 (2.6)	0.54 (0.27–1.07)	0.21
Length of hospitalization (day) (SD)	2.7 (4.1)	2.8 (4.0)	0.04 (−0.47 to 0.55)	0.88
SaO_2_ under room air (%) (SD)	97.4 (2.9)	96.2 (3.0)	1.24 (−1.69 to −0.79)	**<0.001**
Oxygen supplement	28 (5.4)	93 (18.6)	4.04 (2.59–6.30)	**<0.001**
Systemic corticosteroid treatment	42 (8.1)	129 (25.9)	4.23 (2.88–6.20)	**<0.001**
PICU entry	2 (0.4)	4 (0.8)	2.13 (0.39–11.73)	0.39

^*a*^ Multivariable logistic and linear regressions were applied to assess the association between host SNPs and dichotomous and continuous clinical outcomes, respectively, adjusting for age and gender as covariates.

^*b*^ Wheezing illness included asthma exacerbation, acute bronchiolitis, and wheezy bronchitis.

^*c*^ B, β coefficient; OR, odds ratio; PICU, pediatric intensive care unit; RTI, respiratory tract infection; SaO_2_, percutaneous arterial oxygen saturation; SD, standard deviation.

^*d*^ Statistically significant results are highlighted in bold.

### *CDHR3* genotypic association for RV-C susceptibility and clinical outcomes

Table S3 shows tested MAFs of 10 SNPs selected for genotyping. Rs448025 and rs543085868 were previously found to be monoallelic for the major allele in our local children, and they were not analyzed. The genotyping efficiency of eight tagging SNPs were over 95%, and MAFs in our subjects were comparable to those reported for Southern Han Chinese (CHS) in the 1000 Genomes database.

Table 2 summarizes the association between *CDHR3* SNPs and RV-C susceptibility. There was significant association between rs6967330 and RV-C susceptibility (additive model: OR 1.43, 95% CI 1.05–1.93, *P* = 0.021; dominant model: OR 1.45, 95% CI 1.04–2.03, *P* = 0.027). Similarly, the minor allele of rs140154310 was associated with a higher risk of RV-C infection under the additive model (OR 2.53, 95% CI 1.15–5.56; *P* = 0.021).

**TABLE 2:** Associations between RV-C susceptibility and tagging SNPs of *CDHR3* in our children^*c*^

Genotype	All RV-C (*N* = 501)		All RV-A or B (*N* = 518)		*P* value by Pearson *χ* ^2^	Additive model		Dominant model		Recessive model	
	*n*	%	*n*	%		OR (95% CI)	*P* value	OR (95% CI)	*P* value	OR (95% CI)	*P* value
rs3887998											
GG	285	60.1	294	60.1	0.197	1.07 (0.87–1.32)	0.505	1.00 (0.77–1.29)	0.993	1.57 (0.93–2.64)	0.094
GA	152	32.1	170	34.8							
AA	37	7.8	25	5.1							
rs140154310											
CC	467	95.5	487	98.2	**0.017**	**2.53^*d*^ (1.15–5.56)**	**0.021** ^*a*^	ND	ND	ND	ND
CT	22	4.5	9	1.8							
TT	0	0	0	0							
rs73195657											
TT	428	89.2	446	90.1	0.476^*b*^	1.14 (0.77–1.71)	0.516	1.10 (0.73–1.66)	0.650	ND	ND
TC	50	10.4	49	9.9							
CC	2	0.4	0	0							
rs146004234											
GG	460	94.3	483	95.6	0.156^*b*^	1.22 (0.71–2.09)	0.476	1.35 (0.76–2.41)	0.301	ND	ND
GA	28	5.7	20	4.0							
AA	0	0	2	0.4							
rs4730125											
GG	157	41.1	156	40.9	1.000	1.00 (0.82–1.23)	0.982	1.00 (0.75–1.33)	0.974	1.02 (0.69–1.51)	0.930
GT	165	43.2	166	43.6							
TT	60	15.7	59	15.5							
rs6967330											
GG	380	79.5	418	85.0	0.067	**1.43 (1.05–1.93)**	**0.021**	**1.45 (1.04–2.03)**	**0.027**	2.06 (0.62–6.90)	0.241
GA	90	18.8	70	14.2							
AA	8	1.7	4	0.8							
rs73195665											
GG	427	89.3	448	90.5	0.720^*b*^	1.15 (0.77–1.71)	0.496	1.14 (0.75–1.73)	0.547	2.04 (0.18–22.57)	0.561
GA	49	10.3	46	9.3							
AA	2	0.4	1	0.2							
rs408223											
CC	352	81.3	339	78.3	0.524	0.86 (0.64–1.16)	0.328	0.83 (0.60–1.16)	0.272	1.00 (0.35–2.88)	1.000
CG	74	17.1	87	20.1							
GG	7	1.6	7	1.6							

^*a*^ *P* value generated by comparing heterozygous vs homozygous major. Bonferroni correction was employed for the correction of the false discovery rate.

^*b*^ Analyzed by Fisher exact test.

^*c*^ CI, confidence interval; ND, not done; OR, odds ratio; RV, rhinovirus.

^*d*^ Statistically significant results are highlighted in bold.

Table 3 summarized the genotypic associations with outcomes of RV infection. Under the additive model, the minor allele of rs140154310 was associated with higher risk of wheezing illness (OR 2.38, 95% CI 1.12–5.04; *P* = 0.024) after adjusting for age and gender. There was stronger association between this SNP and RV-C-associated wheeze (OR 2.71, 95% CI 1.32–5.58; *P* = 0.007), implying synergistic effect between rs140154310 and RV-C for wheezing. This SNP was also associated with RV-C-associated lower RTI (OR 2.55, 95% CI 1.24–5.24; *P* = 0.011), while the minor allele of rs73195665 was associated with systemic corticosteroid treatment (OR 1.69, 95% CI 1.04–2.75; *P* = 0.035). In addition, the minor allele of rs408223 was inversely associated with RV-C-associated lower RTI (OR 0.67, 95% CI 0.47–0.96; *P* = 0.025). Nonetheless, the top SNP rs6967330 was not associated with any clinical diagnoses and measures of RTI severity.

**TABLE 3:** Associations between tagging SNPs of *CDHR3* and clinical outcomes of our children under the additive model

	Odds ratio (95% CI), *P* value							
SNP (A/a)	Lower RTI	RV-C-associated lower RTI	Wheeze	RV-C-associated wheeze	Asthma exacerbation (AE)	RV-C-associated AE	Oxygen supplement	Systemic corticosteroid
rs3887998	1.01 (0.82–1.24), 0.931	1.09 (0.87–1.36), 0.453	1.00 (0.81–1.23), 0.981	1.04 (0.83–1.30), 0.761	0.98 (0.76–1.25), 0.845	1.03 (0.78–1.35), 0.845	0.79 (0.56–1.12), 0.181	0.97 (0.73–1.29), 0.843
rs140154310	2.06 (0.97–4.37), 0.059	**2.55 (1.24–5.24), 0.011^*a*^**	**2.38 (1.12–5.04), 0.024**	**2.71 (1.32–5.58), 0.007**	1.88 (0.88–4.03), 0.106	1.78 (0.78–4.07), 0.174	0.86 (0.26–2.885), 0.805	1.72 (0.72–4.13), 0.225
rs73195657	1.45 (0.97–2.17), 0.074	1.47 (0.97–2.23), 0.071	1.42 (0.95–2.13), 0.087	1.36 (0.89–2.08), 0.151	1.11 (0.69–1.78), 0.663	0.96 (0.56–1.66), 0.895	1.19 (0.65–2.18), 0.576	1.39 (0.83–2.32), 0.218
rs146004234	0.87 (0.50–1.50), 0.608	1.00 (0.56–1.81), 0.989	0.94 (0.54–1.63), 0.827	1.06 (0.59–1.92), 0.836	0.88 (0.46–1.70), 0.712	1.13 (0.57–2.23), 0.721	0.87 (0.37–2.04), 0.742	1.04 (0.51–2.12), 0.918
rs4730125	0.93 (0.76–1.14), 0.495	0.96 (0.77–1.19), 0.680	0.96 (0.78–1.18), 0.696	0.95 (0.76–1.18), 0.630	0.78 (0.61–1.00), 0.054	0.81 (0.62–1.07), 0.135	0.92 (0.67–1.26), 0.596	0.80 (0.60–1.07), 0.131
rs6967330	1.20 (0.89–1.62), 0.230	1.35 (0.98–1.85), 0.063	1.22 (0.90–1.65), 0.194	1.34 (0.97–1.84), 0.073	1.08 (0.75–1.54), 0.688	1.29 (0.88–1.89), 0.193	1.32 (0.86–2.05), 0.208	1.45 (0.99–2.12), 0.057
rs73195665	1.17 (0.78–1.75), 0.452	1.18 (0.77–1.80), 0.450	1.25 (0.84–1.87), 0.279	1.20 (0.78–1.84), 0.401	1.14 (0.72–1.81), 0.577	1.20 (0.72–1.99), 0.489	0.93 (0.49–1.77), 0.821	**1.69 (1.04–2.74), 0.035**
rs408223	0.93 (0.69–1.25), 0.634	**0.67 (0.47–0.95), 0.025**	0.91 (0.67–1.23), 0.537	0.72 (0.51–1.02), 0.065	1.15 (0.81–1.62), 0.438	0.93 (0.63–1.39), 0.736	1.25 (0.80–1.95), 0.335	1.13 (0.76–1.70), 0.544

^*a*^ Statistically significant results are highlighted in bold.

### Haplotypic association for RV-C susceptibility, diagnosis, and RTI severity

The linkage disequilibrium (LD) plot for the eight SNPs spanning ~10 kbp on *CDHR3* locus revealed two haplotype blocks (Fig. S2). Rs3887998 and rs73195657 defined HapA, while rs4730125, rs6967330, and rs73195665 defined HapB. Table 4 summarizes the results for haplotypic associations. HapB (GAG frequency 0.046) was associated with an increased risk of RV-C infection compared to other species (OR 1.71, 95% CI 1.11–2.65; *P* = 0.016). This risk haplotype was also associated with the need for oxygen supplement (OR 1.93, 95% CI 1.13–3.30; *P* = 0.016). In the meanwhile, HapA (AC frequency 0.050) was associated with lower RTI (OR 1.55, 95% CI 1.02–2.37; *P* = 0.041) but not other clinical parameters. None of the haplotypes was associated with RV-C-associated lower RTI, wheezing illness, and AE.

**TABLE 4:** Haplotypic associations between *CDHR3* SNPs and clinical outcomes from RV infections

Diagnosis	Combination of *CDHR3* SNPs	Haplotype	Frequency	OR (95% CI)	*P* value^*a*^
RV-C infection	rs3887998_rs73195657	GT	0.764	Reference	
		AT	0.182	1.06 (0.84–1.34)	0.625
		AC	0.050	1.07 (0.71–1.61)	0.757
	rs4730125_rs6967330_rs73195665	GGG	0.530	Reference	
		TGG	0.372	1.10 (0.89–1.35)	0.395
		GAA	0.050	1.28 (0.84–1.94)	0.257
		GAG	0.046	**1.71 (1.11–2.65)^*b*^**	**0.016**
Lower RTI	rs3887998_rs73195657	GT	0.764	Reference	
		AT	0.183	0.88 (0.70–1.11)	0.289
		AC	0.050	**1.55 (1.02–2.37)**	**0.041**
	rs4730125_rs6967330_rs73195665	GGG	0.530	Reference	
		TGG	0.372	0.96 (0.78–1.18)	0.685
		GAA	0.050	1.15 (0.76–1.76)	0.512
		GAG	0.046	1.14 (0.75–1.73)	0.554
RV-C-associated lower RTI	rs3887998_rs73195657	GT	0.764	Reference	
		AT	0.182	0.97 (0.75–1.25)	0.822
		AC	0.050	1.53 (1.00–2.35)	0.052
	rs4730125_rs6967330_rs73195665	GGG	0.530	Reference	
		TGG	0.372	1.04 (0.83–1.31)	0.734
		GAA	0.050	1.25 (0.80–1.95)	0.329
		GAG	0.046	1.42 (0.92–2.20)	0.111
Wheeze	rs3887998_rs73195657	GT	0.764	Reference	
		AT	0.183	0.88 (0.70–1.11)	0.386
		AC	0.050	1.49 (0.98–2.27)	0.062
	rs4730125_rs6967330_rs73195665	GGG	0.530	Reference	
		TGG	0.373	0.99 (0.80–1.23)	0.945
		GAA	0.050	1.23 (0.81–1.88)	0.335
		GAG	0.046	1.14 (0.75–1.73)	0.544
RV-C-associated wheeze	rs3887998_rs73195657	GT	0.764	Reference	
		AT	0.183	0.94 (0.73–1.21)	0.633
		AC	0.050	1.39 (0.90–2.14)	0.142
	rs4730125_rs6967330_rs73195665	GGG	0.530	Reference	
		TGG	0.372	1.03 (0.82–1.29)	0.821
		GAA	0.050	1.26 (0.81–1.98)	0.308
		GAG	0.046	1.37 (0.89–2.13)	0.158
Asthma exacerbation	rs3887998_rs73195657	GT	0.764	Reference	
		AT	0.182	0.93 (0.71–1.23)	0.632
		AC	0.050	1.10 (0.68–1.79)	0.687
	rs4730125_rs6967330_rs73195665	GGG	0.530	Reference	
		TGG	0.372	0.79 (0.61–1.03)	0.078
		GAA	0.050	0.93 (0.57–1.52)	0.774
		GAG	0.046	1.02 (0.62–1.67)	0.954
RV-C-associated asthma exacerbation	rs3887998_rs73195657	GT	0.764	Reference	
		AT	0.182	1.06 (0.78–1.43)	0.715
		AC	0.050	0.93 (0.53–1.64)	0.808
	rs4730125_rs6967330_rs73195665	GGG	0.530	Reference	
		TGG	0.372	0.86 (0.64–1.14)	0.289
		GAA	0.050	1.15 (0.68–1.95)	0.610
		GAG	0.046	1.21 (0.72–2.06)	0.470
Oxygen supplement	rs3887998_rs73195657	GT	0.765	Reference	
		AT	0.183	0.68 (0.45–1.01)	0.057
		AC	0.049	1.25 (0.68–2.29)	0.474
	rs4730125_rs6967330_rs73195665	GGG	0.530	Reference	
		TGG	0.371	0.97 (0.69–1.37)	0.876
		GAA	0.051	0.85 (0.42–1.73)	0.655
		GAG	0.046	**1.93 (1.13–3.30)**	**0.016**
Systemic corticosteroid	rs3887998_rs73195657	GT	0.764	Reference	
		AT	0.183	0.83 (0.60–1.16)	0.286
		AC	0.050	1.50 (0.89–2.51)	0.128
	rs4730125_rs6967330_rs73195665	GGG	0.530	Reference	
		TGG	0.372	0.87 (0.64–1.18)	0.373
		GAA	0.050	1.53 (0.91–2.57)	0.108
		GAG	0.046	1.25 (0.73–2.16)	0.420

^*a*^ Adjusted for age and sex as covariates.

^*b*^ Statistically significant results are highlighted in bold.

### Interactions among *CDHR3* SNPs on RV-C susceptibility and clinical outcomes

**Fig 1:**
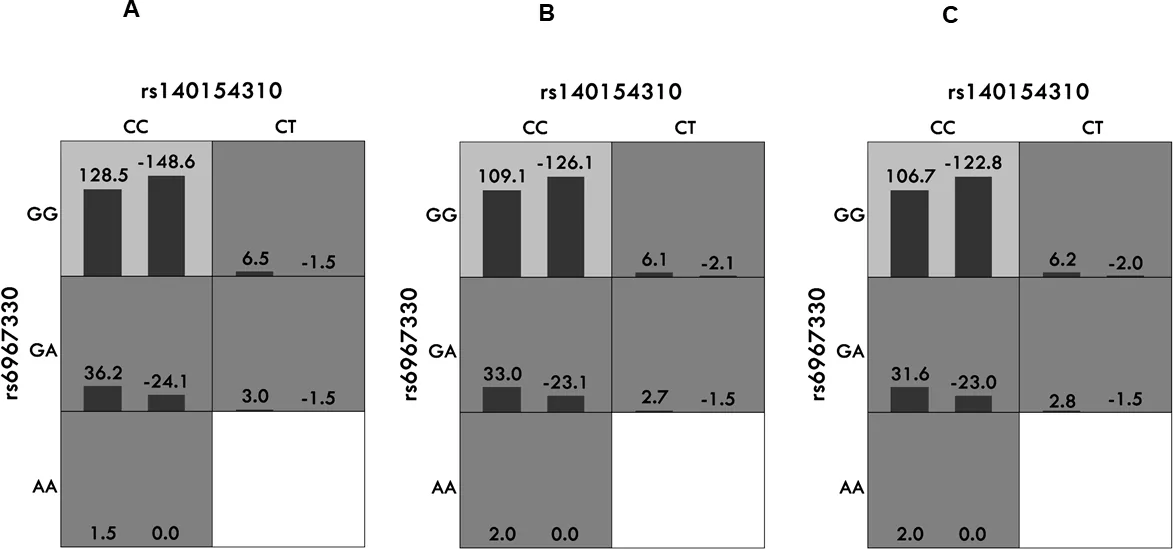
The best two-locus model derived from the GMDR analysis for RV-C-related outcomes. The best two-locus model was composed of SNPs rs6967330 and rs140154310 for (**A**) RV-C infection, (**B**) RV-C-associated lower RTI, and (**C**) RV-C-associated wheezing. Dark shading cells indicate high-risk genotype; light gray cell indicates low-risk genotype; and empty cells are non-shading. No homozygous minor allele exists for rs140154310 in our cohort (Tables 6 and 7). Within each cell, the left bar represents cases with the corresponding RV-C outcomes, and the right bar shows the “control group.” The numbers above bars denote sums of the GMDR scores. Subjects with high-risk genotypes formed by these two SNPs had OR of 1.62 (95% CI 1.17–2.23, *P* = 0.003) for RV-C infection; OR of 1.57 (95% CI 1.13–2.19, *P* = 0.008) for RV-C-associated lower RTI; and OR of 1.56 (95% CI 1.11–2.18, *P* = 0.010) for RV-C-associated wheezing compared with those with low-risk genotypes. GMDR, generalized multifactor dimensionality reduction.

After adjustment for age and gender, GMDR analyses revealed the combination of tagging SNPs rs140154310 and rs6967330 to be the significant model predicting risk for RV-C infections, RV-C-associated lower RTI, and wheezing illness (*P <* 0.05, Table 5; Tables S4 and S5). Having a minor allele at either of these two loci was associated with increased risk for RV-C (OR 1.62, 95% CI 1.17–2.23; *P* = 0.003; Fig. 1A). Similarly, high-risk genotypes were associated with increased risk of RV-C-associated lower RTI (OR 1.57, 95% CI 1.13–2.19; *P* = 0.008; Fig. 1B) and wheezing illness (OR 1.56, 95% CI 1.11–2.18; *P* = 0.010; Fig. 1C) when compared with the low-risk genotype. GMDR analyses also identified a significant four-locus model from rs140154310, rs146004234, rs4730125, and rs6967330 to be associated with increased susceptibility for RV-C (TA > 55%, *P* < 0.001; Table 5). There was no epistatic interaction among the tested SNPs for clinical diagnoses and RTI severity (Tables S6 and S7).

**TABLE 5:** GMDR associations between tagging SNPs of *CDHR3* and RV-C infection^*b*^

Number of locus	SNP combination	CVC	TA (%)	*P* value^*a*^
1	rs6967330	10	54.20	0.005
**2**	**rs140154310, rs6967330^*c*^**	**10**	**55.14**	**0.001**
3	rs140154310, rs146004234, rs6967330	4	55.66	<0.001
**4**	**rs140154310, rs146004234, rs4730125, rs6967330**	**9**	**56.82**	**<0.001**
5	rs3887998, rs140154310, rs146004234, rs4730125, rs6967330	4	52.43	0.198
6	rs3887998, rs140154310, rs73195657, rs146004234, rs4730125, rs6967330	7	50.49	0.445
7	rs3887998, rs140154310, rs73195657, rs146004234, rs4730125, rs6967330, rs408223	4	46.72	0.878
8	rs3887998, rs140154310, rs73195657, rs146004234, rs4730125, rs6967330, rs73195665, rs408223	10	47.36	0.826

^*a*^ Adjusted for age and sex as covariates.

^*b*^ CVC, cross-validation consistency; SNP, single-nucleotide polymorphism; TA, testing accuracy.

^*c*^ Statistically significant results are highlighted in bold.

### Gene-environmental interaction between *CDHR3* SNPs and RV-C infection

Gene-environmental interaction analysis from GMDR found a synergistic relationship between RV-C and *CDHR3_*rs3887998 for more severe RTI infections in terms of oxygen supplement (TA 68.9%, *P* < 0.001; Table 6) and systemic corticosteroid treatment (TA 72.7%, *P* < 0.001; Table 7). Subjects with major allele for rs3887998 and RV-C were at risk of oxygen supplement during hospitalization (OR 4.19, 95% CI 2.67–6.58; *P* < 0.001).

**TABLE 6:** Gene-environmental interactions between RV-C and *CDHR3* SNPs on requirement for oxygen supplement^*b*^

Number of factors	Combination	CVC	TA (%)	*P* value *^a^*
**1**	**RV-C**	**10^*c*^**	**67.95**	**<0.001**
**2**	**RV-C, rs3887998**	**10**	**68.92**	**<0.001**
3	RV-C, rs3887998, rs408223	5	68.38	<0.001
4	RV-C, rs3887998, rs140154310, rs408223	4	69.98	<0.001
5	RV-C, rs3887998, rs4730125, rs73195665, rs408223	4	62.61	0.003
6	RV-C, rs3887998, rs140154310, rs4730125, rs73195665, rs408223	5	63.91	0.001
7	RV-C, rs3887998, rs73195657, rs4730125, rs6967330, rs73195665, rs408223	7	62.11	0.004
**8**	**RV-C, rs3887998, rs140154310, rs73195657, rs4730125, rs6967330, rs73195665, rs408223**	**10**	**62.24**	**0.005**
**9**	**RV-C, rs3887998, rs140154310, rs73195657, rs146004234, rs4730125, rs6967330, rs73195665, rs408223**	**10**	**61.07**	**0.006**

^*a*^ Adjusted for age and sex as covariates.

^*b*^ CVC, cross-validation consistency; SNP, single-nucleotide polymorphism; TA, testing accuracy.

^*c*^ Statistically significant results are highlighted in bold.

**TABLE 7:** Gene-environmental interactions between RV-C and *CDHR3* SNPs on systemic corticosteroid treatment^*b*^

Number of factors	Combination	CVC	TA (%)	*P* value^*a*^
**1**	**RV-C**	**10^*c*^**	**71.86**	**<0.001**
2	RV-C, rs73195657	7	71.60	<0.001
3	RV-C, rs73195657, rs4730125	5	71.51	<0.001
**4**	**RV-C, rs3887998, rs73195657, rs4730125**	**10**	**72.67**	**<0.001**
**5**	**RV-C, rs3887998, rs73195657, rs4730125, rs73195665**	**8**	**69.34**	**<0.001**
**6**	**RV-C, rs3887998, rs73195657, rs4730125, rs6967330, rs73195665**	**8**	**69.27**	**<0.001**
7	RV-C, rs3887998, rs140154310, rs73195657, rs4730125, rs6967330, rs73195665	7	69.58	<0.001
**8**	**RV-C, rs3887998, rs140154310, rs73195657, rs4730125, rs6967330, rs73195665, rs408223**	**8**	**67.15**	**<0.001**
**9**	**RV-C, rs3887998, rs140154310, rs73195657, rs146004234, rs4730125, rs6967330, rs73195665, rs408223**	**10**	**64.41**	**<0.001**

^*a*^ Adjusted for age and sex as covariates.

^*b*^ CVC, cross-validation consistency; SNP, single-nucleotide polymorphism; TA, testing accuracy.

^*c*^ Statistically significant results are highlighted in bold.

## DISCUSSION

This case-control study extended the knowledge of *CDHR3* by reporting a new risk SNP rs140154310 and its epistatic interaction with the known risk variant rs6967330 on increased susceptibility and severity of RV-C-associated RTIs. We identified genotypic and haplotypic associations between rs140154310 and rs6967330 for RV-C susceptibility. These factors also interacted to confer increased risk of wheezing illness from RV infection and requirements for oxygen supplement and systemic corticosteroid treatment.

CDHR3 is the RV-C-specific surface receptor on airway epithelial cells, while the ubiquitous ICAM-1 and LDLR are the receptor for RV-A and RV-B (10). Rs6967330-A was consistently reported to be a risk allele for early-onset severe asthma and more frequent wheezing and RTIs, particularly those caused by RV-C infections (9, 11 – 13). Compared with the dominant rs6967330-G (Cys529), the asthma risk allele rs6967330-A (Tyr529) led to higher surface expression of transmembrane protein CDHR3, whereby increasing RV-C binding and replication (6). In contrast to the intuitive assumption that rs6967330-A was the late mutation that predisposed asthmatics to RV-C infections, evolutionary studies suggested that this allele was the ancient ancestral allele (14). Rs6967330-G was the more modern missense mutant that selected out under the pressure of evolving RV-C species to protect the general population from this viral infection by encoding the defective CDHR3 protein and downregulating its surface expression (15, 16).

Apart from replicating the known association between RV-C and rs6967330, our data suggested that *CDHR3*_rs140154310, an intron SNP adjacent to the top SNP rs6967330, synergistically contributed to RV-C susceptibility and severity of wheezing. The main genetic variants of *CDHR3* differed across populations (17). Among our Chinese subjects, rs140154310 was associated with increased risk of RV-C-associated RTI. This SNP was also involved in the two-locus GMDR model for RV-C susceptibility and RV-C-associated lower RTI and wheezing (Table 5; Tables S4 and S5). The previous study from our team also reported rs140154310 to be associated with more frequent wheezing and diminished lung function indices in Chinese preschool children (13). However, *in silico* prediction indicated low probability of this mutant for regulating protein transcript splicing (13).

In our current cohort with RV-associated RTIs, we identified genotypic and haplotypic associations between *CDHR3* risk genotypes and RV-C infection and extended this genetic association to the requirement for oxygen supplement. Subgroup analyses showed that children with risk genotype infected with RV-C had even higher risks of wheezing illness (OR increased from 2.38 to 2.71) and oxygen supplement (OR from 4.04 to 4.19) by gene-environmental interaction analysis. The missense variant rs6967330 of *CDHR3* increased RV-C susceptibility by upregulating surface expression of RV receptor CDHR3 in ciliated airway epithelium (7 – 9, 18). We hypothesize that the synergistic effect between *CDHR3* SNPs on RV-C susceptibility and more severe RTIs is mediated through regulation of surface expression of trans-membrane CDHR3 protein. Experimental plasmid transfection of *CDHR3* with rs140154310 and rs6967330 mutants into Hela cell lines could be the first step to understand the effect of rs140154310-T on transcription (mRNA) and surface expression (protein immunofluorescence) of the RV-C receptor CHDR3. Alternatively, we could measure CDHR3 mRNA level in peripheral blood or *in vitro* culture of epithelial cells from wheezing children who carried the two *CDHR3* mutants so that we could analyze their correlations with the severity of wheezing illnesses.

Our study suggested that *CDHR3* haplotype block with rs4730125, rs6967330, and rs73195665 (GAG) was associated with RV-C susceptibility and requirement of oxygen supplement. Moreover, GDMR analysis revealed epistatic interaction between CDHR3 SNPs and RV-C infection on the severity of RTIs. More recent evidence suggested genetic and environmental risk factors combined to determine the presenting RTI phenotype. In asthma development, host and environmental microbiota modulated the host immunity along with early-life allergen exposures to affect hosts’ anti-viral immune responses, airway remodeling and inflammation, and asthma development (19). Host risk genotype also played a pivotal role in this intricate interaction. However, it was unclear how the genetic expression was regulated by environmental stimuli. Hammar et al. described decreased expression of *CDHR3* mRNA in the peripheral blood leukocytes of preschool children with RV-associated wheezing, particularly among those with the risk A allele at *CDHR3*_rs6967330 (20). Forno and coworkers found an epigenetic prediction model based on DNA methylation of *CDHR3* and other asthma genes to distinguish between atopic and non-atopic phenotypes in school-age asthmatic children (21). Further investigations that integrate *CDHR3* risk haplotype, epigenetic profile, resulting mRNA expression, RV-C infection, and clinical severity indices might help to identify genotype prediction model for asthma and RTI severity in paediatric patients.

Furthermore, the human body functions as a sophisticated entirety with complicated interplay networks, so a single SNP or gene cannot be the sole determinant for severity of RV-associated RTIs. On the other hand, different genes would interact with each other to regulate anti-viral immunity. The locus 17q21 was widely recognized as an asthma gene cluster that regulated airway remodeling and chemokine responses (22). RV stimuli increased the expression of *ORMDL3* and *GSDMB* on 17q21 locus in peripheral blood mononuclear cells (23). More recently, Eliasen et al. identified interaction between *CDHR3* and *GSDMB* in children with early-onset severe asthma and homozygous G allele at *GSDMB*_rs2305480 (24). They further reported that this interaction might be mediated through upregulation of anti-viral cytokine IL-17A in peripheral blood. However, they did not detect any interaction between *CDHR3* and *GSDMB* for RV-C susceptibility.

This study had several limitations. Firstly, RV titer could not be measured in the archived NPA samples. It remains unknown whether higher RV-C viral load mediated more severe RTIs. Secondly, the genotypes of other asthma genes were not determined in our cohort, so we were unable to examine any gene-gene interaction outside the *CDHR3* locus. Future prospective studies with larger samples size and freshly collected respiratory samples are required to validate our findings of gene-gene and gene-environment interactions for the severity of RV-associated RTIs.

## MATERIALS AND METHODS

### Study population and design

This cross-sectional study retrieved nasopharyngeal aspirates (NPAs) archived in microbiology laboratories of two public hospitals under the New Territory East cluster in Hong Kong. These NPA samples were subjected for RV and host *CDHR3* genotyping. The relationship between severity of RTIs and genetic and environmental factors were examined. The medical records of children aged below 18 years hospitalized for RTIs in these two hospitals between January 2015 and December 2016 were screened. On each calendar day, the first two enterovirus/rhinovirus-positive cases were selected to avoid seasonality bias. This study excluded subjects with co-morbidities that affected the assessment of clinical severity of RV infections such as compromised immunity, aspiration pneumonia, and unremitted bronchopulmonary dysplasia and co-infection with other respiratory pathogens.

### Rhinovirus genotyping

As a standard practice, NPA was collected within 24 hr from patients hospitalized for RTIs. Following multiplex respiratory virus detection, the remaining NPA samples were stored at −80°C until analysis (2). Details on RV genotyping were described in the online supplement.

### SNP selection and genotyping of *CDHR3*

Tagging SNPs of *CDHR3* were selected using tagging algorithm of HaploView v.5.0 (Broad Institute, Cambridge, MA), following our published criteria (13). Briefly, the studied SNPs had MAF of ≥0.01 and *r* ^2^ of ≥0.8 for LD in CHS as cited in the 1000 Genomes database. The selection region covered 5 kbp both upstream and downstream from rs6967330, with this top SNP being forced to be included in the tagging process. Host genomic DNAs extracted from archived NPA samples were subjected to *CDHR3* genotyping by TaqMan SNP Genotyping Assays (Applied Biosystems) using QuantStudio 12K Flex Real-Time PCR System (Applied Biosystems).

### Assessment of clinical outcomes

Two paediatricians independently reviewed the medical records for patients’ discharge diagnoses and RTI severity. The exclusion criteria for clinical severity analyses included (i) medical records with insufficient details for the severity of RTIs; (ii) co-infection with other viral or bacterial pathogens; (iii) co-morbidities predisposing to more severe outcomes, such as unremitted bronchopulmonary dysplasia, recurrent aspiration pneumonia, and immunocompromised conditions (congenital immunodeficiency, long-term treatment with systemic corticosteroids, and chemotherapy) within 3 months.

Respiratory diagnoses were categorized into (i) upper RTI, (ii) wheezing illness, and (iii) lower RTI. The severity of RTIs was assessed based on duration of hospitalization, transcutaneous oxygen saturation level, requirement for oxygen supplement, treatment with systemic corticosteroid, admission to pediatric intensive care unit, and requirement for assisted breathing (see online supplement).

### Statistical analysis

Demographic and clinical characteristics were compared by *t*-test or analysis of variance for continuous variables and Pearson’s *χ* ^2^ test or Fisher exact test for categorical variables respectively. Hardy-Weinberg equilibrium for selected SNPs was evaluated with *χ* ^2^ exact test. Multivariable logistic and linear regression was used to analyze the association between *CDHR3* SNPs and dichotomous and continuous clinical outcomes, adjusting for age and gender as covariates. Bonferroni correction was used to adjust for multiple statistical comparisons. Haploview v.5.0 (Daly Lab, Cambridge, MA) (25) was applied to identify haplotypes and calculate pairwise LD coefficients. Associations between haplotypes and clinical outcomes were analyzed by multivariate regression using R package *haplo.stats* (https://www.r-project.org/) (26).

GMDR was used to examine gene-gene interactions among SNPs and gene-environmental interaction between SNPs and RV-C infections (27). Empirical *P* values from GMDR prediction models were estimated by comparing the observed average prediction error to the distribution of average prediction error under the non-association null hypothesis from 5000 permutations. Models of maximized prediction accuracy were selected for ≥8/10 cross-validation consistency (CVC) and ≥0.55 testing accuracy (TA). Similar to multivariable regression, haplotype association and GMDR analyses were also adjusted for age and gender as covariates. Statistical comparisons were made two tailed using SPSS v.28 (IBM Corporation, Chicago, IL), with nominal significance level set at 0.05.

## SUPPLEMENTAL MATERIAL

Online Tables and Figures spectrum.01181-23-s0001.docx Tables S1-S7 and Figures S1-S3.

ASM does not own the copyrights to Supplemental Material that may be linked to, or accessed through, an article. The authors have granted ASM a non-exclusive, world-wide license to publish the Supplemental Material files. Please contact the corresponding author directly for reuse.
