# Supplementary material for: Interactive effects between CDHR3 genotype and rhinovirus species for diagnosis and severity of respiratory tract infections in hospitalized children
Source: Microbiol Spectr. 2023 Sep 26;11(5):e01181-23. doi: 10.1128/spectrum.01181-23 (PMC10581227; doi:10.1128/spectrum.01181-23)
Supplement: Online Tables and Figures — Tables S1-S7 and Figures S1-S3. [file spectrum.01181-23-s0001.docx]

**Interactive Effects between *CDHR3* Genotype and Rhinovirus Species for Diagnosis and Severity of Respiratory Tract Infections in Hospitalized Children**

Yu P. Song^1^*, Man F. Tang^1,2^*, Agnes S. Y. Leung^1,2^, Kin P. Tao^1,2,3^, Oi M. Chan^1^, Gary W. K. Wong^1^, Paul K. S. Chan^4^, Renee W. Y. Chan^1,2,3^, Ting F. Leung^1,2,3^

^1^Department of Pediatrics, The Chinese University of Hong Kong, Prince of Wales Hospital, Hong Kong

^2^Hong Kong Hub of Pediatric Excellence, The Chinese University of Hong Kong, Hong Kong

^3^The Chinese University of Hong Kong-University Medical Center Utrecht Joint Research Laboratory of Respiratory Virus and Immunobiology, The Chinese University of Hong Kong, Hong Kong

^4^Department of Microbiology, The Chinese University of Hong Kong, Prince of Wales Hospital, Hong Kong

* Equal contributions as co-first authors

**Correspondence:**

Ting Fan Leung, Room 84043, Lui Che Woo Clinical Sciences Building, Prince of Wales Hospital, Shatin, New Territories, Hong Kong

Tel: (852) 3505 2981. Fax: (852) 2636 0020. E-mail: [tfleung@cuhk.edu.hk](mailto:tfleung@cuhk.edu.hk)

**Table S1. Demographic and clinical characteristics between cases with and without co-infections**

|  | Respiratory co-infection  (N=311) | RV only  (N=1019) | *P*-value |
| --- | --- | --- | --- |
| Age, year (SD) | 3.3 (2.9) | 3.1 (4.6) | 0.49 |
| Male (%) | 59.2 | 62.1 | 0.35 |
| SNP rs6967330 (%) |  |  | 0.84 |
| Homozygous major (GG) | 83.0 | 81.8 |  |
| Heterozygous (AG) | 18.0 | 17.0 |  |
| Homozygous minor (AA) | 1.0 | 1.3 |  |
| RV genogroups |  |  | **< 0.001** |
| RV-A | 161 (51.8) | 477 (46.8) |  |
| RV-B | 34 (10.9) | 41 (4.0) |  |
| RV-C | 116 (37.3) | 501 (49.2) |  |
| Co-morbidities (%) | 39 (12.5) | 151 (14.8) | 0.96 |
| Malignancy (intact immune) | 3 | 17 |  |
| Metabolic, neurologic or genetic | 16 | 51 |  |
| Cardiovascular | 9 | 31 |  |
| Respiratory | 3 | 11 |  |
| Renal | 3 | 17 |  |
| Gastrointestinal | 2 | 6 |  |
| Non-malignant hematologic | 3 | 18 |  |
| Risk factors for asthma* (N) | 85 | 418 |  |
| Personal history of allergy (%) | 37.7 | 37.3 | 0.96 |
| Family history of allergy (%) | 66.7 | 66.5 | 0.99 |
| Smoking exposure (%) | 35.5 | 46.4 | 0.26 |
| Positive API (%) | 64.7 | 59.1 | 0.53 |

API, asthma predictive index.

* Only cases with recurrent wheezing were evaluated for asthma risk factors.

**Table S2. Rhinovirus species and frequency of co-infections**

|  | RV-A  (N=638) | RV-B  (N=75) | RV-C  (N=617) | *P*-value |
| --- | --- | --- | --- | --- |
| Respiratory viruses detected in NPA (%) (N=277)* | 137  (21.5) | 33  (44.0) | 107  (17.3) | **< 0.001** |
| RSV | 28 | 3 | 15 |  |
| Adenovirus | 41 | 11 | 32 |  |
| Influenza | 22 | 3 | 17 |  |
| Parainfluenza | 37 | 11 | 29 |  |
| Coxsackie virus | 6 | 4 | 8 |  |
| *Mycoplasma pneumoniae* | 10 | 5 | 6 |  |
| Herpes simplex virus | 0 | 1 | 4 |  |
| EBV | 1 | 0 | 0 |  |
| Enterovirus, Picornavirus | 1 | 0 | 2 |  |
| Bacterial infection^†^ (%) | 69 (10.8) | 7 (9.3) | 39 (6.3) | **0.014** |
| Respiratory specimen | 25 | 2 | 11 |  |
| Urine | 21 | 2 | 12 |  |
| Blood | 2 | 0 | 0 |  |
| Stool | 20 | 0 | 14 |  |
| Others | 1 | 3 | 2 |  |

NPA, nasopharyngeal aspirate; RV, rhinovirus; RSV, respiratory syncytial virus; EBV, Epstein-Barr virus.

* The sum of co-infected viruses was greater than 277 because some cases had more than one non-RV pathogens detected in NPA.

^†^ Positive bacterial culture from respiratory and other specimens collected during hospitalization.

**Table S3. The details and minor allele frequencies of tagging SNPs of *CDHR3***

| SNP | Position | Cytogenetic band | Nature of SNP | Major allele | Minor allele | MAF of CHS from 1000 Genomes | MAF in current cohort | |
| --- | --- | --- | --- | --- | --- | --- | --- | --- |
|  |  |  |  |  |  |  | RV-C | RV-A or B |
| rs3887998 | 105653867 | 7q22.3 | Intron | G | A | 0.210 | 0.238 | 0.225 |
| rs140154310 | 105653880 | 7q22.3 | Intron | C | T | 0.033 | 0.022 | 0.009 |
| rs73195657 | 105654609 | 7q22.3 | Intron | T | C | 0.071 | 0.056 | 0.049 |
| rs146004234 | 105656263 | 7q22.3 | Intron | G | A | 0.024 | 0.029 | 0.024 |
| rs4730125 | 105656840 | 7q22.3 | Intron | G | T | 0.371 | 0.373 | 0.373 |
| rs6967330 | 105658451 | 7q22.3 | Missense  (C529Y) | G | A | 0.071 | 0.111 | 0.079 |
| rs73195665 | 105659249 | 7q22.3 | Intron | G | A | 0.024 | 0.055 | 0.048 |
| rs448025 | 105660496 | 7q22.3 | Intron | T | G | 0.024 | ND | ND |
| rs408223 | 105662024 | 7q22.3 | Intron | C | G | 0.129 | 0.102 | 0.117 |
| rs543085868 | 105662156 | 7q22.3 | Intron | A | C | 0.019 | ND | ND |

SNP, single nucleotide polymorphism; MAF, minor allele frequency; CHS, southern Chinese Han population; ND, not done.

All genotyped SNPs of all subjects were in Hardy-Weinberg equilibrium with *P* > 0.05.

**Table S4. GMDR associations between tagging SNPs of *CDHR3* and RV-C-associated lower RTI**

| Number of locus | SNP combination | CVC | TA (%) | *P*^†^ |
| --- | --- | --- | --- | --- |
| 1 | rs6967330 | 10 | 54.27 | 0.009 |
| **2** | **rs140154310, rs6967330** | **10** | **54.88** | **0.004** |
| 3 | rs140154310, rs6967330, rs408223 | 4 | 56.32 | 0.003 |
| 4 | rs73195657, rs4730125, rs6967330, rs408223 | 3 | 52.30 | 0.218 |
| 5 | rs3887998, rs73195657, rs4730125, rs6967330, rs408223 | 9 | 51.68 | 0.306 |
| 6 | rs3887998, rs140154310, rs73195657, rs4730125, rs6967330, rs408223 | 4 | 50.37 | 0.465 |
| 7 | rs3887998, rs140154310, rs73195657, rs146004234, rs4730125, rs6967330, rs408223 | 10 | 49.91 | 0.505 |
| 8 | rs3887998, rs140154310, rs73195657, rs146004234, rs4730125, rs6967330, rs73195665, rs408223 | 10 | 49.47 | 0.569 |

SNP, single nucleotide polymorphism; CVC, cross-validation consistency; TA, testing accuracy.

^†^ Adjusted for age and sex as covariates.

**Table S5. GMDR associations between tagging SNPs of *CDHR3* and RV-C-associated wheezing**

| Number of locus | SNP combination | CVC | TA (%) | *P*^†^ |
| --- | --- | --- | --- | --- |
| 1 | rs6967330 | 10 | 54.06 | 0.012 |
| **2** | **rs140154310, rs6967330** | **10** | **54.70** | **0.007** |
| 3 | rs140154310, rs6967330, rs408223 | 4 | 56.12 | 0.004 |
| 4 | rs3887998, rs73195657, rs4730125, rs6967330 | 9 | 53.81 | 0.099 |
| 5 | rs3887998, rs73195657, rs4730125, rs6967330, rs408223 | 7 | 50.88 | 0.404 |
| 6 | rs3887998, rs140154310, rs73195657, rs4730125, rs6967330, rs408223 | 5 | 51.52 | 0.326 |
| 7 | rs3887998, rs140154310, rs73195657, rs146004234, rs4730125, rs6967330, rs408223 | 9 | 51.85 | 0.283 |
| 8 | rs3887998, rs140154310, rs73195657, rs146004234, rs4730125, rs6967330, rs73195665, rs408223 | 10 | 50.37 | 0.458 |

SNP, single nucleotide polymorphism; CVC, cross-validation consistency; TA, testing accuracy.

^†^ Adjusted for age and sex as covariates.

**Table S6. GMDR associations between tagging SNPs of *CDHR3* and clinical diagnoses**

| Number of locus | SNP combination | CVC | TA (%) | *P*^†^ |
| --- | --- | --- | --- | --- |
| Associations between tagging SNPs and lower RTI | | | | |
| 1 | rs73195657 | 5 | 51.83 | 0.092 |
| 2 | rs73195657, rs408223 | 4 | 52.45 | 0.130 |
| 3 | rs3887998, rs4730125, rs6967330 | 4 | 50.07 | 0.503 |
| 4 | rs3887998, rs73195657, rs4730125, rs6967330 | 7 | 49.83 | 0.526 |
| 5 | rs3887998, rs73195657, rs4730125, rs6967330, rs408223 | 9 | 47.20 | 0.824 |
| 6 | rs3887998, rs73195657, rs146004234, rs4730125, rs6967330, rs408223 | 4 | 48.34 | 0.732 |
| 7 | rs3887998, rs140154310, rs73195657, rs146004234, rs4730125, rs6967330, rs408223 | 7 | 47.84 | 0.776 |
| 8 | rs3887998, rs140154310, rs73195657, rs146004234, rs4730125, rs6967330, rs73195665, rs408223 | 10 | 46.09 | 0.905 |
| Associations between tagging SNPs and wheezing | | | | |
| 1 | rs73195657 | 5 | 51.74 | 0.108 |
| 2 | rs73195657, rs6967330 | 4 | 53.50 | 0.036 |
| 3 | rs3887998, rs4730125, rs6967330 | 3 | 50.11 | 0.495 |
| 4 | rs3887998, rs73195657, rs4730125, rs6967330 | 7 | 52.46 | 0.195 |
| 5 | rs3887998, rs73195657, rs146004234, rs4730125, rs6967330 | 7 | 54.46 | 0.050 |
| 6 | rs3887998, rs73195657, rs146004234, rs4730125, rs6967330, rs408223 | 6 | 49.56 | 0.573 |
| 7 | rs3887998, rs140154310, rs73195657, rs146004234, rs4730125, rs6967330, rs408223 | 9 | 48.73 | 0.673 |
| 8 | rs3887998, rs140154310, rs73195657, rs146004234, rs4730125, rs6967330, rs73195665, rs408223 | 10 | 46.59 | 0.879 |
| Associations between tagging SNPs and asthma exacerbation | | | | |
| 1 | rs4730125 | 10 | 53.72 | 0.108 |
| 2 | rs73195657, rs4730125 | 10 | 53.16 | 0.170 |
| 3 | rs3887998, rs4730125, rs73195665 | 2 | 49.36 | 0.594 |
| 4 | rs3887998, rs73195657, rs4730125, rs73195665 | 8 | 51.25 | 0.366 |
| 5 | rs3887998, rs73195657, rs4730125, rs6967330, rs73195665 | 5 | 53.13 | 0.180 |
| 6 | rs3887998, rs73195657, rs4730125, rs6967330, rs73195665, rs408223 | 7 | 50.01 | 0.509 |
| 7 | rs3887998, rs73195657, rs146004234, rs4730125, rs6967330, rs73195665, rs408223 | 7 | 50.05 | 0.507 |
| 8 | rs3887998, rs140154310, rs73195657, rs146004234, rs4730125, rs6967330, rs73195665, rs408223 | 10 | 50.44 | 0.448 |

SNP, single nucleotide polymorphism; CVC, cross-validation consistency; TA, testing accuracy.

^†^ Adjusted for age and sex as covariates.

**Table S7. GMDR associations between tagging SNPs of *CDHR3* and severity of RV-associated RTI**

| Number of locus | SNP combination | CVC | TA (%) | *P*^†^ |
| --- | --- | --- | --- | --- |
| Associations between tagging SNPs and oxygen supplement | | | | |
| 1 | rs6967330 | 9 | 52.38 | 0.251 |
| 2 | rs3887998, rs73195665 | 5 | 52.37 | 0.281 |
| 3 | rs3887998, rs73195657, rs73195665 | 3 | 55.60 | 0.081 |
| 4 | rs3887998, rs73195657, rs4730125, rs6967330 | 9 | 50.36 | 0.473 |
| 5 | rs3887998, rs73195657, rs4730125, rs6967330, rs73195665 | 4 | 50.73 | 0.449 |
| 6 | rs3887998, rs73195657, rs4730125, rs6967330, rs73195665, rs408223 | 7 | 50.09 | 0.497 |
| 7 | rs3887998, rs73195657, rs146004234, rs4730125, rs6967330, rs73195665, rs408223 | 8 | 47.71 | 0.705 |
| 8 | rs3887998, rs140154310, rs73195657, rs146004234, rs4730125, rs6967330, rs73195665, rs408223 | 10 | 42.33 | 0.947 |
| Associations between tagging SNPs and systemic corticosteroid treatment | | | | |
| **1** | **rs6967330** | **8** | **55.57** | **0.011** |
| 2 | rs140154310, rs6967330 | 4 | 56.78 | 0.002 |
| 3 | rs3887998, rs4730125, rs73195665 | 6 | 52.50 | 0.280 |
| 4 | rs3887998, rs73195657, rs4730125, rs73195665 | 9 | 53.44 | 0.201 |
| 5 | rs3887998, rs73195657, rs4730125, rs73195665, rs408223 | 9 | 55.55 | 0.082 |
| 6 | rs3887998, rs140154310, rs73195657, rs4730125, rs73195665, rs408223 | 4 | 54.27 | 0.152 |
| 7 | rs3887998, rs140154310, rs73195657, rs4730125, rs6967330, rs73195665, rs408223 | 9 | 49.85 | 0.525 |
| 8 | rs3887998, rs140154310, rs73195657, rs146004234, rs4730125, rs6967330, rs73195665, rs408223 | 10 | 47.17 | 0.747 |

SNP, single nucleotide polymorphism; CVC, cross-validation consistency; TA, testing accuracy.

^†^ Adjusted for age and sex as covariates.

**Figure S1. Flowcharts of sample inclusion and exclusion**

Chinese children aged **0-18** years old with EV/RV infection who were hospitalized in pediatric wards of two public hospitals during Jan 2015 to December 2016

*Two NPA randomly identified and retrieved per calendar day*

**1564** NPAs retrieved for nucleic acid extraction and RV genotyping

**1523** NPAs had conclusive results for both host *CDHR3* SNPs and RV genotyping

Exclusions:

- 16 person times of repeat hospitalization*;
- 177 cases with co-morbidities predisposing to more severe respiratory outcomes^†^;
- 311 cases excluded for co-infection with non-RV respiratory pathogens

**1019** cases of RV-associated RTIs analyzed for the associations between *CDHR3* genotype, RV species, and clinical severity

EV/RV, enterovirus/rhinovirus (by molecular assay); NPA, nasopharyngeal aspirate; RTI, respiratory tract infection.

* Repeat hospitalization indicates two NPAs from the same individual on two separate admissions during the study period.

^†^ Co-morbidities include unremitted bronchopulmonary dysplasia, recurrent aspiration pneumonia and immunocompromised conditions (congenital immunodeficiency, long term treatment with systemic corticosteroids and chemotherapy) within three months.

**Figure S2. Linkage disequilibrium pattern plot of *CDHR3* SNPs**


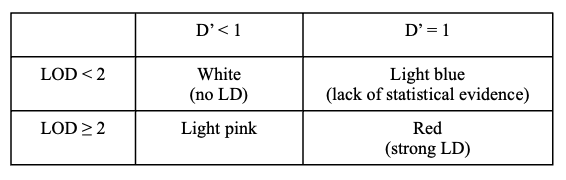

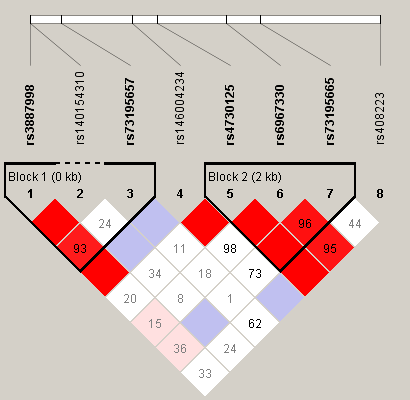


LD, linkage disequilibrium; LOD, log of the likelihood odds ratio.

Analysis result of Haploview (v5.0), with the reference track on top indicating SNP positions on *CDHR3* locus. The strength of linkage between polymorphic markers was displayed in different colors (see box). The block encircled within the triangle represented regions of high LD. Numbers within the boxes demonstrated the coefficients of LD (D’) between the polymorphic markers.

**Figure S3. The best two-factor model for oxygen requirement derived from the GMDR analysis**


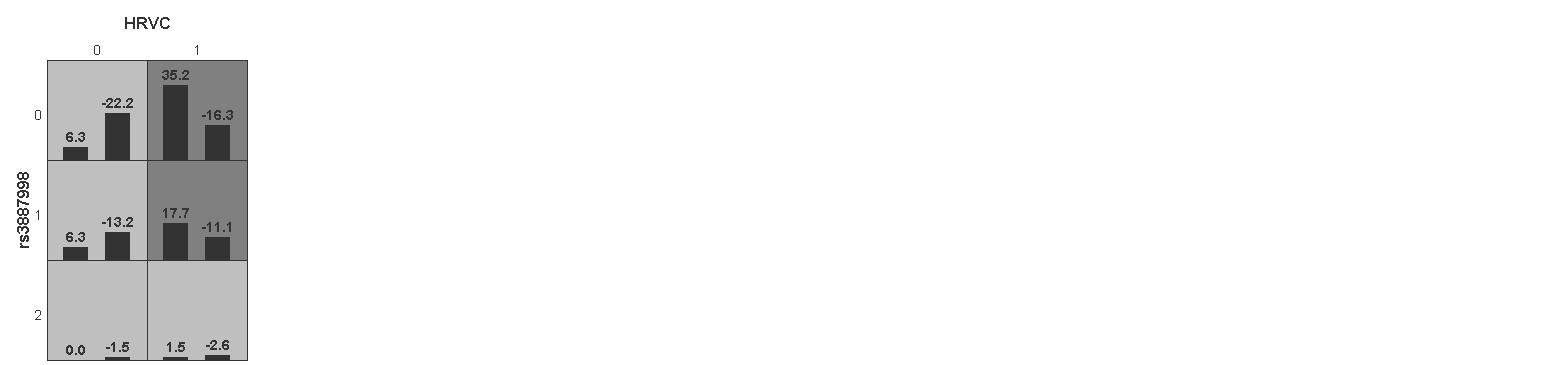


The best two-factor model from gene-environmental interaction GMDR analysis for oxygen supplementation was composed of RV-C infection and SNP rs3887998. Dark shading cells indicate high-risk group, light grey cell indicates low-risk group, and empty cells are non-shading. Within each cell, the left bar represents cases requiring oxygen supplements, and the right bar indicates those not requiring oxygen. The numbers above bars denote sums of the GMDR scores. Subjects with high-risk genotypes and RV-C infection had an odds ratio 4.19 (95% CI 2.67-6.58; ***P*<0.001**) for oxygen supplementation compared with those in low-risk group. GMDR, generalized multifactor dimensionality reduction.
